# Supplementary material for: Real-World Effectiveness of the mRNA COVID-19 Vaccines in Japan: A Case–Control Study
Source: Vaccines (Basel). 2022 May 14;10(5):779. doi: 10.3390/vaccines10050779 (PMC9145554; doi:10.3390/vaccines10050779)
Supplement: Supplementary file 1 [file vaccines-10-00779-s001.zip › Table S1_r1.pdf]

Table S1. Adjusted odds ratio (aOR) against COVID-19 according to diagnosis period, age group, and vaccine manufacture by place of care or stay.

		Diagnosis period				Age group				Manufacturer			
	Vaccine	June and July		August and September		<60 years old		≥60 years old		Pfizer		Moderna	
	doses	aOR*	95% CI	aOR*	95% CI	aOR*	95% CI	aOR*	95% CI	aOR*	95% CI	aOR*	95% CI
All cases	0	1	(Reference)	1	(Reference)	1	(Reference)	1	(Reference)	1	(Reference)	1	(reference)
	1	0.38	(0.07-2.10)	0.32	(0.17-0.60)	0.34	(0.19-0.62)	0.19	(0.03-1.31)	0.38	(0.21-0.71)	0.36	(0.12-1.10)
	2	0.08	(0.01-0.65)	0.21	(0.11-0.40)	0.24	(0.12-0.46)	0.06	(0.01-0.37)	0.19	(0.10-0.36)	0.21	(0.05-0.87)
Hospitalized cases	0	1		1		1.00		1		1.00		1	
	1	n.c		0.20	(0.06-0.61)	0.14	(0.04-0.46)	n.c		0.17	(0.05-0.52)	0.22	(0.04-1.23)
	2	n.c		0.02	(0.003-0.149)	0.04	(0.01-0.25)	n.c		0.02	(0.004-0.11)	0.08	(0.003-1.98)
Stay at hotel or home cases	0	1	(Reference)	1	(Reference)	1.00	(Reference)	1	(Reference)	1.00	(Reference)	1	(reference)
	1	0.12	(0.006-2.39)	0.30	(0.16-0.58)	0.38	(0.20-0.71)	0.09	(0.01-1.13)	0.40	(0.21-0.77)	0.39	(0.12-1.33)
	2	0.005	(<0.0011-0.21)	0.26	(0.13-0.50)	0.29	(0.14-0.60)	0.03	(0.003-0.35)	0.23	(0.11-0.45)	0.24	(0.05-1.10)

*Sex, age (10-year intervals), area of residence, underlying medical conditions, PCR test date, handwashing for 20 s, current alcohol drinking, live in a single-family home, and family members commute to work or school. n.c means not calculated.
