# Supplementary figures and images for: Real-World Effectiveness of the mRNA COVID-19 Vaccines in Japan: A Case–Control Study
Source: Vaccines (Basel). 2022 May 14;10(5):779. doi: 10.3390/vaccines10050779 (PMC9145554; doi:10.3390/vaccines10050779)

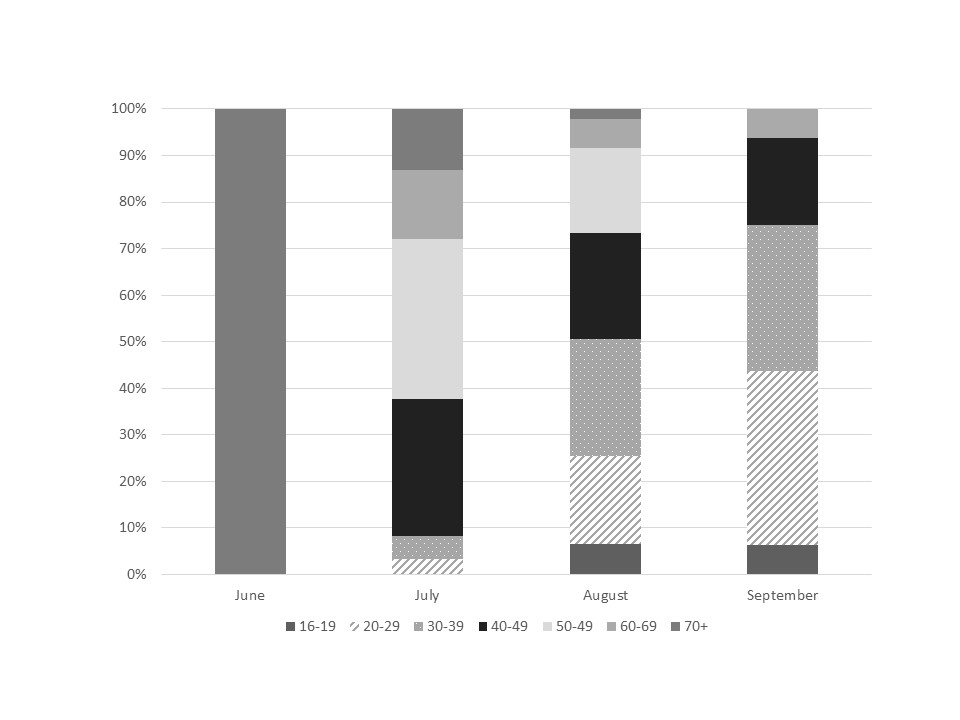

Supplement: Supplementary file 1 [file vaccines-10-00779-s001.zip › Figure S1_r1.jpg]
